# EEG in Education: A Scoping Review of Hardware, Software, and Methodological Aspects

**DOI:** 10.3390/s25010182

**Published:** 2024-12-31

**Authors:** Christos Orovas, Theodosios Sapounidis, Christina Volioti, Euclid Keramopoulos

**Affiliations:** 1 Department of Products and Systems Design Engineering, University of Western Macedonia, 50100 Kozani, Greece; 2 School of Philosophy and Education, Aristotle University of Thessaloniki, 54124 Thessaloniki, Greece; teo@edlit.auth.gr (T.S.); chvolioti@gmail.com (C.V.); 3 Department of Information and Electronic Engineering, International Hellenic University, 57001 Nea Moudania, Greece; euclid@ihu.gr

**Keywords:** electroencephalogram (EEG), neuroeducation, brain signals, event-related potentials (ERP)

## Abstract

Education is an activity that involves great cognitive load for learning, understanding, concentrating, and other high-level cognitive tasks. The use of the electroencephalogram (EEG) and other brain imaging techniques in education has opened the scientific field of neuroeducation. Insights about the brain mechanisms involved in learning and assistance in the evaluation and optimization of education methodologies according to student brain responses is the main target of this field. Being a multidisciplinary field, neuroeducation requires expertise in various fields such as education, neuroinformatics, psychology, cognitive science, and neuroscience. The need for a comprehensive guide where various important issues are presented and examples of their application in neuroeducation research projects are given is apparent. This paper presents an overview of the current hardware and software options, discusses methodological issues, and gives examples of best practices as found in the recent literature. These were selected by applying the PRISMA statement to results returned by searching PubMed, Scopus, and Google Scholar with the keywords “EEG and neuroeducation” for projects published in the last six years (2018–2024). Apart from the basic background knowledge, two research questions regarding methodological aspects (experimental settings and hardware and software used) and the subject of the research and type of information used from the EEG signals are addressed and discussed.

## 1. Introduction

Almost a century after the first recording, EEG has provided precious information about the activity of cerebral neurons for scientific fields such as neuroscience and cognitive science. The technological developments in the area have provided portability and wireless connectivity to the EEG devices and they have facilitated the use of the necessary apparatus outside of medical laboratory settings. Thus, brain signals are being used for applications in human–computer interaction (HCI) to control devices with brain–computer interfaces (BCI) and examine the responses of the brain while performing various cognitive tasks.

Learning is a cognitive process which results in a change of behavior and acquisition of new knowledge [1]. It is a lifelong process that helps organisms adapt to their environment. Education is an organized approach to learning where specific tasks, goals, methods, materials, and interactions are involved. Whether an educational method is successful or not it is a question of how well students perform in assessment procedures related to the taught materials. What happens within their minds though is a research question of psychology, cognitive science, educational science, and neuroscience.

The combination of the above fields has given rise to neuroeducation, or educational neuroscience, or ‘mind, brain, and education’ [2] for studying the activities occurring in the brain when individuals learn, and applying this knowledge to improve teaching practices and optimize educational methods [3]. Although the gap between the neuronal functions and education might be too far to be the subject of an interdisciplinary field, as some early skepticism mentioned [4], there are quite a few examples where cognitive neuroscience has been successfully combined with behavioral data analysis in order to complement and extend existing knowledge of the relevant cognitive processes [2].

Brain imaging methods such as electroencephalogram (EEG), magnetoencephalogram (MEG), functional magnetic resonance imaging (fMRI), and functional near infrared spectroscopy (fNIR) have provided the necessary tools to study the operation of the brain while performing cognitive tasks. EEG is based on recording mainly the postsynaptic electrical activity of pyramidal cerebral neurons, which are almost vertical to the cerebral surface [5]. MEG records the related electromagnetic field alterations from the electrical activity of the brain [6]. The fMRI [7] and fNIR [8] are based on measuring the blood oxygenation levels alterations in the brain based on magnetic and optical absorption characteristics, respectively. All of the above methods register signals from the continuous operation of the brain. These signals convey information about the time and the place where the action is taking place. However, apart from MEG, which has both detailed time and space resolution, EEG is very precise about the time things are happening but lacks space resolution, while fMRI and fNIR have very high space resolution but lack specific time resolution since blood oxygen level variations do not happen concurrently with the corresponding neuronal activity. While spatial information is very important to determine the functional and structural relations of brain areas, precise time details are also very important, especially when event-related potentials (ERPs) are involved, as explained later. As far as mobility is concerned, EEG and fNIR are the basic methods which can operate out of laboratory settings, while portable MEG systems based on optical pumped magnetometers (OPMs) are still in their early stages [9]. As far as cost is concerned, EEG is the most competitive method for limited budget projects.

The above characteristics of EEG have made this brain imaging method the main tool for research in cognitive science and related areas and scientific fields. As mentioned above, neuroeducation is such a field. Brain signals can be used to obtain insights on cognitive processes, providing connections between their occurrences and the signal features appearing at the same time. Functional brain connectivity is a very important aspect to reveal coordinated brain activity, and different approaches, including deep learning, can be used to estimate coherence among neuronal areas [10]. Mental states during learning tasks, concentration, and engagement, and arousal levels along with brain synchronization between the people involved are only some of the key performance indicators that can be used. This form of brain–computer interfacing is defined as passive BCI in [10] and regards the analysis of the brain activity of the user without any target monitoring. However, the two other forms of BCI according to [10], active and reactive, can be used. The former is related to the use of changes in the brain activity, directed from the neurointerface operator, interpreted as controlling commands and it is closer to the general idea of BCI. The latter detects and classifies brain responses to external stimulation. Passive and reactive BCIs seem to be closer to the needs of neuroeducation.

Regarding the use of biomedical information in education, a systematic review of wearable biosensor technology in education was recently made available [11]. A descriptive and bibliometric study focused on educational neuroscience is also available in [12]. As there is a large variety of research methodologies, metrics, scenarios and circumstances, and research targets that can be used in neuroeducation, a comprehensive guide describing basic issues and presenting current neuroeducation research practices is useful for entering this field from various disciplines. This is the motivation behind this scoping review which is focused on EEGs, and the research questions that are addressed in this paper are the following:

**Q1.**  *What are the methodological aspects that are used by the research projects in neuroeducation in terms of experimental settings such as sample sizes, hardware, and software used?*

**Q2.**  *What is investigated in neuroeducation research projects? What is the subject of their research and what information is extracted and used from the EEG signals?*

The rest of the paper is organized as follows: Section 2, Section 3 and Section 4 provide some necessary background knowledge before Q1 and Q2 can be addressed. More specifically, Section 2 presents information regarding the information that can be extracted from EEGs and the necessary steps. Section 3 outlines the hardware aspects of EEG devices and Section 4 discusses the software aspects and options that exist. Section 5 provides the methodology which was followed for the selection of the papers and the protocol for their review. The results and discussion based on Q1 and Q2 are presented in Section 6. This paper concludes by summarizing the presented issues and discussing future trends in the field.

## 2. Information from EEGs

Neurons are the cells of the neural system that are related to coding and transmitting information. There are about 10^11^ neurons in the human brain and each one of them can communicate with up to 10 thousand other neurons. In their generic form, neurons consist of (i) their cell body, (ii) the dendrites which receive chemical messages from other cells, and (iii) an axon which transfers messages to other neurons, muscles, or glands. At the end of each neuron’s axon there can exist many endings which are connected to the dendrites of other neurons. These connections are called synapses, and they operate electrochemically using chemical substances which are called neurotransmitters. These can control the ion gates regulating the input and output flow of charged ions, thus creating electrical potentials. The *action* potential is created and propagates within the neuron from its dendrites to its axon endings and the *postsynaptic* potential (PSP) is the one which is created at the neurons’ inputs (dendrites). Although at a smaller scale from the action potential, the PSP (~10 mV) appears in a more synchronized basis with other neurons and has a longer duration (~30 ms). This is why the PSP is considered the main source of the currents which are recorded from the EEG [5]. More specifically, the recordings of EEGs come from the activity of the cerebral cortex neurons which have pyramidal form and are almost vertical to its surface. Since the human body is filled with electrically conductive material, these currents can be transferred to the outer surface of the scalp where they are recorded using the EEG electrodes [13].

### 2.1. Brain Waves

**Figure 1:**
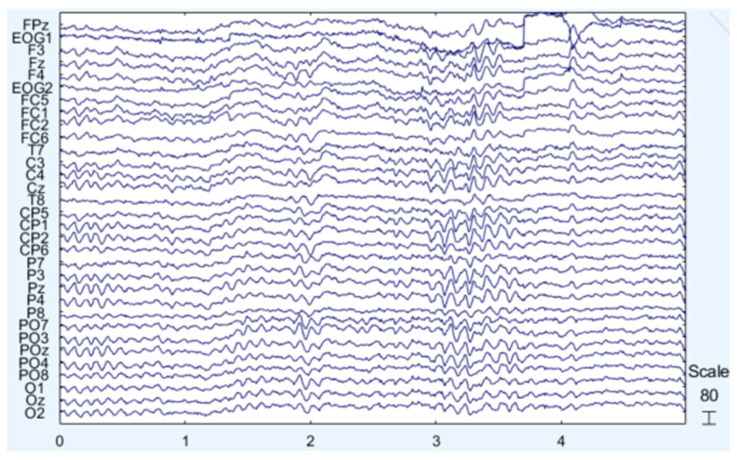
Example of 5 s EEG recording (source: sample data plot from EEGLAB [14]). There are 32 channels, with their naming derived from the 10–20 system, and the scale refers to microvolts (μV).

As mentioned, the signals which are recorded in EEG come from the PSPs of the neurons in the cerebral cortex. The amplitude and the frequency of these signals are related to the actions and the states of the brain (e.g., sleep, awake, active, concentrated, etc.). There are five basic bands of the human brain waves. In ascending frequency order, they are the following [13]: (i) δ (delta) from 0.5 to 4 Hz. These are usually related to deep sleep although they can also be measured in awakened subjects. (ii) θ (theta) from 4 to 7.5 Hz. These appear in relaxation states, drowsiness, and deep meditation. (iii) α (alpha) from 8 to 13 Hz. These are an indication of a relaxed state without focusing son something specific and they are usually recorded in the occipital lobe area (where the visual cortex lies) when subjects have their eyes closed. In this band, but mainly in frontal areas of the brain, in the motor cortex, the μ (mu) waves can be also recorded in relaxed states without movements. (iv) β (beta) from 14 to 26 Hz. These are related to concentration and focusing on solving a problem. (v) γ (gamma) from 30 to 45 Hz. These are not very frequent in normal conditions and they have low amplitudes. An example of a five second EEG recording is shown in Figure 1.

The study of the characteristics of brain waves and their distribution on the scalp has created a link between them and specific states of mind. The degree of correlation or synchronization among signals from different electrodes can be a measurement of connectivity patterns [15]. Alpha band intensity ratios have been studied as indexes of mental workload where EEG features based on alpha-to-theta and theta-to-alpha ratios have provided high classification accuracy [16]. Engagement, relaxation, interest, and focus are measured from EEG signals in [17], based on characteristic features extracted from the relations between the bands [18]. The use of a high gamma band as a correlate for engagement is proposed in [19]. Frontal alpha asymmetry (FAA), defined as an alpha activity difference between the right and left hemispheres of the brain, serves as a biomarker for motivation and affect [20] and it is also linked to exaggerated physiological arousal [21] or avoidance and negation [22].

### 2.2. Event-Related Potentials

One of the greatest advantages of EEGs compared to other brain imaging methods is its very fine time resolution. Brain activity is recorded by its electrical representation at the very exact time instant that it happens [23]. Every time there is a stimulus that is perceived by the brain, a series of mental processes begin to analyze it and prepare a possible reaction. The corresponding electrical activity variations which are recorded in the EEG are called evoked potentials (EP). When the stimulus is related to higher cognitive functions that are taking place in the cerebral cortex and they are related to sensory, cognitive, affective, or motor processes, these potentials are called event-related potentials (ERP) and they can serve as a measure of the brain activity [24].

The basic characteristic of ERPs is the time dependance on the events they relate to and they are thus time-locked with the events. The time in which they appear is called latency. Their intensity and their spatial distribution also characterize them, although the spatial resolution of EEGs is poor. That is, EEGs can tell exactly when something happened in the brain, but an indication about the location must be derived after a series of data processing. ERPs are EEG fluctuations that are observed after an event and the polarity and latency of their signal variation can be used for their naming (e.g., a negative peak at 170 msec after the event is called N170). The time of the event is time 0. When latency is greater than 100, ERPs are related to the cognitive processing which is evoked and they are characterized as endogenous [13]. ERPs are considered the result of brain electrical activity in a collection of areas of the cerebral cortex where equivalent electrical dipoles correspond to the electrical postsynaptic activity of sets of neurons [25]. That is, the ERPs which are recorded by the electrodes which are placed in specific locations of the scalp come from the combination of currents created in various areas of the brain. An additional advantage of ERPs is that not only the time but also what is happening in the cerebral cortex in-between the event and the resulting action can be revealed [26].

As mentioned already, an initial naming convention for the ERPs was derived from the polarity of the greatest peak in the signal and its latency. Thus, there are ERPs with names such as P300, P3, N170, N400, etc. Some of the most well-known ERPs which are used in neurocognitive research are the following:

N170: This mainly relates to optical discrimination, face and familiar objects recognition [27,28]. N250 is also of a similar kind [29]. These ERPs are also called N1 and N2 and they also relate to inhibition in control tasks along with the P3 ERP that we present below.

N400: This relates to responses to meaningful content in a flow of words that we read [30,31].

P3 (or P300): This relates to optical and audio oddballs, and it is mainly traced in occipital lobe areas. The frequency of the oddball is in inverse analogy with the amplitude of the signal [32,33].

MMN (mismatch negativity): This is similar to P300 but requires no attention from the participant. It is associated with acoustic operation for oddball recognition and it is observed around 150–200 ms after the stimuli over fronto-central regions of the brain [34].

ERN (error-related negativity): This is related to the acknowledgment of a wrong choice [35]. It is a negative variation of about 100 ms after the incorrect movement. It is mainly observed around the frontal lobe.

Reward positivity (RewP): This is a positive waveform appearing in frontal to medial lobe areas in around 200–300 ms after the presentation of positive feedback or the outcome of an action [36]. It is related to ERN in the sense that it can be considered absent or suppressed following non-reward [37].

The above ERPs, along with many others (C1, N2pc, LRP, SSVEP, etc.), are the basic elements in applications such as brain–computer interfaces (BCI) and research in neuroeducation. The main problem in their use is the many preprocessing steps needed for their isolation, as these EEG variations (1–30 μV) are observed together with the usual recorded brain activity (~100 μV). These issues are discussed in the next subsections.

### 2.3. EEG Analysis Steps

Due to the small scale of the currents that are being recorded and the various interferences that may exist, the raw EEG multichannel signal which is recorded by the electrodes may be contaminated with noise and artifacts of various frequencies and forms. Thus, a series of processing and analysis steps is necessary before any information can be extracted from the EEG.

High-pass filtering with a cutoff frequency of 0.5 Hz and low-pass filtering with a cutoff frequency in the range of about 50–70 Hz is the first step. The ubiquitous 50 Hz (up to 60 Hz) signal due to the electrical power network can be eliminated with notch filters. Artifacts can be due to physiological or systemic factors. Physiological artifacts can be due to body movement and muscle activity, movements of the eyes or electrocardiogram (ECG) signal, and sweating. Systemic artifacts are power supply interference, cable defects, electrical noise, and unbalanced impedances of the electrodes.

A recent presentation of the denoising methods and other processing steps in the pipeline of EEG signal analysis can be found in [38,39] and it is thoroughly presented in [40]. Linear regression, blind source separation (BSS), independent components analysis (ICA), principal components analysis (PCA), canonical correlation analysis (CCA), wavelet transform (WT), and empirical mode decomposition (EMD) are the main methods that are used [40]. The basic principle in most of them is the decomposition of the EEG signals, the detection and removal of contaminated parts and artifacts, and the reconstruction of the signals.

The downsampling of the data to obtain files of smaller sizes which facilitate the computer analysis can be performed as well. Signal acquisition can take place at rates as high as 1024 Hz, and thus a conversion to 256 or 128 Hz would reduce sizes four or six times, respectively.

After the above initial preprocessing steps, a feature extraction procedure needs to take place [15,41]. Joint time frequency analysis can be applied to data epochs to reveal the frequencies of the brain waves and their intensities in function with time. Segmentation in epochs is also very useful for extracting ERP information. The triggering events are usually located at time 0 and a time window before and after this instant is included in the epoch. These epochs are then averaged together per channel to give a clear view of the ERP under examination.

Both classical signal analysis techniques and machine learning (ML) tools can be used for the classification and detection of patterns of neuronal activity during specific cognitive tasks. Representation learning using deep learning [42] can be used to transform raw data into representations of feature vectors needed for classification providing adequate levels of tolerance to accommodate variability among the users.

## 3. Hardware Aspects

Down to its main components, a digital EEG device consists of the sensors, an amplifier and filtering unit, an analog to digital converter (ADC), and a communication unit. The EEG used in neuroeducation is non-invasive and electrodes are attached externally, on the surface of the skin. However, regarding other uses of EEG in brain–machine interfaces (BMI), and especially for rehabilitation purposes, there is also the possibility for implanted electrodes, either on the surface of the brain (electrocorticography, EcoG) or even on the outer 1 to 2 mm of the cortex (intracortical) [43]. Due to the external placement of the electrodes on the skin of the scalp, the voltages that are recorded are in the range of 1–100 μV and this is a crucial factor in most applications that use EEG. This is because extra care must be taken in the correct placement of the electrodes.

Since the outer layer of the skin is a bad conductor of electricity, a gel might need to be used in order to lower the impedance between the skin and the electrode [44]. Although improving the signal to noise ratio (SNR), the use of gel is time consuming and not easy, especially when the EEG recordings should be in classroom conditions rather than laboratory settings. The need to use the equipment in a more flexible and fast manner has made space for the development of portable, low-cost, and consumer-grade devices which can also communicate wirelessly with the signal acquisition computer. The existence of these devices has really made a revolution in the field of EEG research for non-medical purposes. A crucial factor for the existence of low-cost portable devices is the development of dry electrodes which allows faster and easier preparation. In addition to the two electrode options mentioned above (gel or dry), there is also the use of wet electrodes, which is a compromise between the discomfort but better conduction of the gel electrodes, and the flexibility and fast placement of the dry ones. However, the use of dry electrodes seems to be achieving high standards, obtaining comparable results to wet electrodes [45]. Another categorization of electrodes is whether they are active or passive. In the former case, they coexist with a pre-amplifier so that the signal is directly amplified at the source before it is sent to the collecting module. The latter does not use preamplification at the electrode level. Although this can be important for the quality of the signal, the final performance also depends on other parameters [44].

EEG recording can be bipolar or unipolar. In the first case, electrodes are paired, and their potential differences are recorded. In the unipolar case, the recorded potential difference is between an electrode and a reference electrode which is common between more electrodes. Electrodes can be placed on the head attached to an elastic cap following the standardized 10–20 set of positioning and naming [46], fixed on an elastic head strap, or fixed on a specially made *spider*-like plastic base. The first option is the standard method and has preset positions where 128 or more electrodes can be attached, although usually fewer are used. The second option is used by smaller scaled systems of two to four electrodes and the third option can be used with up to 14–20 electrodes.

Although there are ongoing efforts to build open and do-it-yourself EEG devices, such as the openEEG project in [47], the more convenient case is to use portable, wireless, low-cost consumer-grade devices. A very recent and complete survey of such EEG systems is presented by Niso et al. in [44]. A previous similar review exists in [48]. As it will be observed from the presentation of some recent neuroeducation projects in the next section as well, the most common low-cost devices which are reportedly used in the literature are from the Emotiv (Epoc-X and Epoc-flex), openBCI (Ganglion and Cyton + Daisy), Neurosky (Mindwave), InteraXon (Muse 2 and Muse S), and Macrotellect (Brainlink Pro) companies. With a cost of either less than USD 1000 or up to USD 2000, they are an affordable and decent solution, especially in cases where many of them must be used concurrently to measure the brain activity of groups of students in classroom settings. Ear-centered devices have also been presented (e.g., enophones) and they appear to be an alternative to classical cap EEG recording for auditory attention monitoring [49]. Medium to high cost devices are also being used according to the budget of the project and the EEG recording requirements (e.g., Brain Products ActiCAP, ActiCHamp, LiveAmp, and BIOPAC’s MP150 and B-Alert).

## 4. Software Aspects

**Figure 2:**
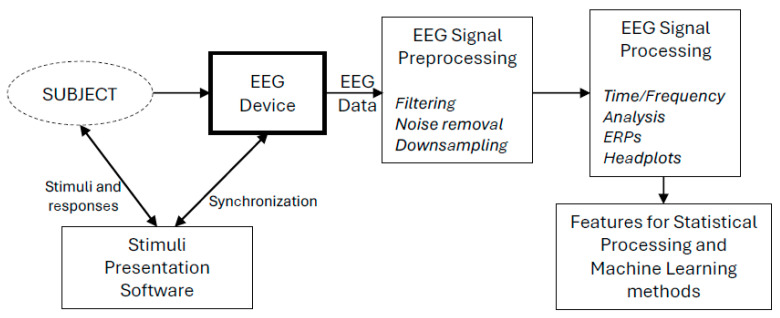
The conceptual framework for the usage of software in EEG applications.

The role of the software used in EEGs is the management and analysis of the EEG data. A conceptual framework of its usage is depicted in Figure 2.

As depicted in Figure 2, EEG data are being recorded from a subject using an EEG device. During this process, stimuli could be presented to the subject (e.g., images, sounds, etc.) and responses (e.g., the pressing of buttons) could be received as well. These stimuli and responses should be synchronized with the EEG device so that EEG data will include the time signatures of the events and responses. The data coming from the EEG device need to be analyzed, as already mentioned in Section 2.3. This analysis includes preprocessing, where noise removal, filtering, and downsampling can be performed, and processing, where time/frequency characteristics of the signals are retrieved, ERPs are analyzed, and information is produced so that it can plotted in 2D and 3D head plots. After these steps, feature extraction can take place so that further analysis can be performed using statistical or machine learning methods.

All of the digital equipment which can be used for EEG recording, from the least expensive to the highest quality medical devices, is accompanied by the relevant proprietary software which offers a specific range of processing, viewing, and storing options. However, the lack of compatibility in file formats and the limitation of data processing to the options provided by that software was an inhibitory factor in EEG research [50].

With the gradual emergence of open source EEG processing software and data standardization, the research community has been able to develop, modify, and use a variety of processing tools through these software packages [51]. Depending on processing requirements, user interface preferences, working environment, and familiarity with programming, the options that exist nowadays are plenty. Some of these are presented below.

EEGLAB [14]. This is an integrated software package running on MATLAB (The Mathworks, Inc., Natick, MA, USA). It provides functionality both through the command line and a graphical user interface (GUI). It supports the processing of raw EEG data and provides a set of ERP processing options. It can apply ICA, and create topographic maps of wave and energy distribution on the head following time and frequency analysis, artefacts detection, etc. The main EEGLAB reference article ([14]) has almost 22,000 citations (December 2023) and maybe this fact by itself describes the great acceptance of this package. It provides levels of functionality, starting with command line functions and reaching GUI interaction for all operations. EEGLAB supports many datafile types (e.g., set, edf, edf+, bdf, gdf, mff, cnt, and eeg) and its open architecture allows the creation of many plugins (~150) with many additional functions and enhanced data handling. EEGLAB can also run on Octave [52] which is an open software alternative to MATLAB. Although EEGLAB can run on Octave, this compatibility is not yet guaranteed for its extensions, such as ERPLAB which is an EEGLAB plugin presented next. However, due to the grant-privileged pricing options for academia [51], the use of MATLAB as a basis for software packages can be beneficial in academic research.

ERPLAB [53]. As mentioned above, this is an EEGLAB plugin. Its aim is to provide extra functionality for ERP processing, visualization, and analysis. It offers enhanced filtering capabilities, reference point alteration, data preprocessing, epoching, and artifact detection.

Corrmap [54] is also a MATLAB toolbox for performing ICA and a semi-automatic clustering of independent components to artifacts and brain-related signals from EEG recordings.

OpenViBE [55]. This is an open software platform that provides functionalities for designing brain–computer interfaces, interfacing them with virtual environments and enabling the visual feedback of brain activity in real time. It has a set of modules for the acquisition, preprocessing, main processing, and visualization of EEG data that can be interconnected according to the user’s requirements using a graphical interface. The user can also add new modules according to their requirements as a software development kit (SDK) in C++ is provided.

Fieldtrip [56]. This is a software toolkit that runs on top of MATLAB and can be used to analyze data derived from MEG, EEG, and other biosignals. It does not use a GUI but provides a large set of high- and low-level functions. The former are used by users while the latter are largely hidden from them.

BCIPy [57]. This is Python-based software focused on research in brain–computer interface systems using ERP interfacing for text dictation. It can also be of more general use and supports data acquisition, stimulus presentation, data search, signal processing and visualization, and language modeling and uses a simple graphical user interface.

MNE [58]. This is a software package in Python that provides analysis and workflow tools for preprocessing, source localization, analysis in the time and frequency domains, statistical analysis, and methods for estimating functional connectivity between brain regions.

BIOSIG [50]. An open-source software library for processing a variety of biomedical signals such as those from the EEG, electrocorticalogram (ECoG), electrocardiogram (ECG), electroophthalmogram (EOG), electromyogram (EMG), etc. Tools are provided for many applications, such as BCIs, neurophysiology, psychology, etc. Options are also given for data acquisition, artefact processing, quality control, feature extraction, categorization, modeling, visualization, etc. Originally for MATLAB and Octave, it also enables use with C++, Python, Java, and R.

PsychoPy [59]. An application for creating experiments which offers ease of use due to its graphical interface and flexibility due to the availability of Python scripts. In comparison with the previously mentioned software tools, PsychoPy is not concerned with EEG processing. Its main role is to conduct experiments in behavioral sciences and neuroimaging experiments. It can present stimuli on a computer screen and communicate with the EEG device, which is being used, to send a “trigger”. This is especially useful in experiments involving ERPs where the exact time of the stimuli onsets must be recorded along with the corresponding EEG data. The trigger signals can also be sent by network connections allowing for remote recording and experimentation.

Stimulus presentation can also be created and controlled by Psychophysics (Psychtoolbox-3) which is a MATLAB toolbox fully compatible with Octave [60,61].

The above were some of the software options which can be employed in experiments involving EEGs, such as the ones in neuroeducation. These are mainly open software solutions, while at the same time, the proprietary software which comes with the EEG devices can offer a range of processing and analysis tools.

## 5. Methodology

**Figure 3:**
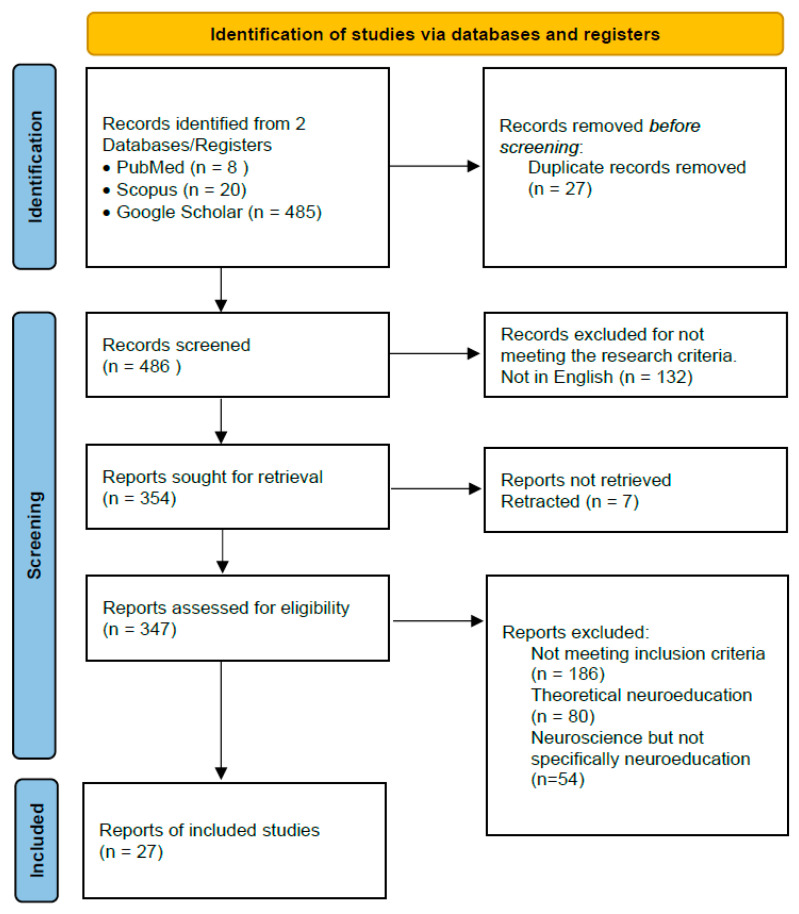
The PRISMA flow diagram.

As mentioned in the introduction, the purpose of this paper is to provide a comprehensive guide and survey to the current state of the multidisciplinary field of neuroeducation by examining relevant projects based on using EEGs. The kind of information that can be extracted from the brain signals and the relevant hardware and software options were discussed in the previous sections to provide an initial background knowledge so that projects related to neuroeducation can be presented next. Thus, recent neuroeducation research projects, as found in the literature by searching in PubMed, Scopus, and Google Scholar with the keywords “EEG and neuroeducation”, are presented in the following section focusing on their methodological aspects regarding Q1 and Q2. The Preferred Reporting Items for Scoping Reviews and Meta-Analyses (PRISMA) statement [10] was followed for the selection of the papers and the corresponding flow diagram is depicted in Figure 3.

The search was focused on neuroeducation projects published in the last six years (2018–2024). The initial screening of the papers was based on their keywords and abstracts. A focus on neuroeducation using EEGs, a clear presentation of research targets and methodological issues, and a detailed explanation of the research procedures regarding hardware, software, and samples used were the basic criteria for the inclusion of a paper in this review. Research projects also had to be published in peer reviewed journals or international conferences. PubMed returned 8 papers and 6 of them were included in the review. Scopus returned 20 papers and 13 of them were included in the review. Google scholar returned 485 results and 8 of them were included in the review as they were not in the results of the previous databases.

## 6. Results and Discussion

This section briefly presents, initially, a collection of recent and characteristic neuroeducation projects focusing on their methodological characteristics as well as the hardware and software options that were used. Further analysis and discussion then follow based on Q1 and Q2. The numbers before the paragraphs are used for indexing to the corresponding lines of Table 1, where a number of characteristic details for each project are presented.

1. The efficacy of Just in Time Teaching in anatomy education, where pre-class exercises are used by instructors to establish foundational knowledge in novice learners and focus on more complex concepts, was tracked using EEGs in [62]. Two ERPs were used as metrics in that research. The first was N250 and the second was RewP (reward positivity). As presented earlier, N250 relates to object recognition and increases in amplitude with perceptual expertise [29]. RewP is associated with the evaluation of performance feedback [36] and decreases with the enhancement of evaluating the correctness of a response as the subject learns. The above ERP behaviors were observed in the experiments with 24 (11 m*ale* + 23 f*emale*) participants of 20.74 years mean age (m. age), where computer-based learning modules were used. The Psychophysics Toolbox extensions [60,61] and a customized MATLAB script were used for questions presented on a computer screen. The ActiCAP XPress EEG with 16 ch V-Amp system (Brain Products, GmbH, Munich, Germany) and Brain Vision Recorder Software (Version 1.20, Brain Products, GMbH, Munich, Germany) were employed for EEG acquisition.

2. ERPs were also used as metrics in [63], where the level of diagnostic accuracy in images of bovine pathology was under examination as to whether it was the result of image recognition or the acquisition of diagnostic expertise. The ERPs that were used were RewP and N170. As mentioned above, RewP is associated with the evaluation of performance feedback. N170 is related to optical discrimination and the recognition of faces, familiar objects, or words [27]. According to the study in [63], while the amplitude of RewP decreased with learning in novices it remained significantly different from the corresponding ERP in experts, where it did not change throughout the course of the experiment. Regarding N170, there was no change in the signals from the novices and they also remained significantly lower in amplitude compared to those of the experts. MATLAB and the Psychophysics Toolbox extensions were again used for displaying the questions to the subjects as in [62] and the ActiCAP slim with 16 ch V-Amp EEG system (Brain Products, GmbH, Munich, Germany) and Brain Vision Recorder Software (Version 1.20, Brain Products, GMbH, Munich, Germany) were again employed for EEG acquisition. Twenty-six undergraduate students (23 f. + 3 m.) were recruited as novices with a mean age of 19.8 years. Six female and three male experts with a mean age of 44.1 years were recruited as experts.

3. The negative value of the means of EEGs, in addition to the time needed to perform programming tasks, was used as a metric in a study on the brain activity of computer science students in visual and textual programming [64,65]. The BIOPAC MP150 data acquisition unit and AcqKnowledge 4.3 Software were used for data acquisition, analysis, storage, and retrieval. Eight volunteers, first-year computer science students, were recruited (3 f. and 5 m.) and the research was carried out in university labs in a properly designed place. The negative value of the means of EEGs is considered to indicate a higher cognitive response [66], and that is the reason that it was used as a metric in this study. The need for differentiated education programs depending on the learning skills and cognitive abilities of the students was stated in this study. Also, the invariance of the programming language used was also reflected in the results. The small number of students for the experiments and the necessity for special places to perform the EEG measurements were mentioned as limitations of this study.

4. Another study which is related to computer programming learning and knowledge transfer is presented in [67]. The relationship between cognitive load and prior knowledge, as indicated by working memory usage, was investigated in a project with eight male (eight m.) participants, 18–21 years old, which were about to learn the C programming language. Their prior programming knowledge varied from beginner to average, creating two groups: sufficient previous knowledge (SPK) and insufficient previous knowledge (IPK). EEG data were collected using the EPOC-X device while the participants were completing 17 learning tasks related to computer programming and presented with the OpenSesame software. EEGLAB was used for filtering and ICA. The spatio-temporal brain data (SPBD) were provided to NeuCube software to create neural connections and connectivity patterns using computational models based on Spike Neural Networks (SNNs). Percentages of the brainwave bands theta and alpha during the learning tasks, related to cognitive load, were also considered. The research demonstrated that neuronal connectivity and spike activity patterns as visualized in NeuCube can be used to understand and interpret brain activity patterns during the transfer learning process

5. Brain synchronization and engagement was under investigation in [68] where the inter-subject correlation (ISC) [69] was measured in EEGs recorded by 42 female students of m. age = 22.4 watching videos in a classroom. The feasibility of performing ISC using low-cost comfortable and affordable equipment such as the Smartphone Brain Scanner [70] which uses a modified version of Emotiv EPOC+ was successfully demonstrated. EEG processing was performed with the Corrmap plug-in [54] for EEGLAB [14].

6. Spatial reasoning in children was the subject of the study in [71], where the learning of the law of reflection was examined using the N2 and P3 ERPs and joint time frequencies (JFTs) of alpha waves at sites F3, F4, and Pz while learning through video game play. These neural correlates, together with behavioral measures of performance in learning the concepts, were investigated in 21 children (11 boys and 10 girls) from 6 to 12 years old. EEG recording was performed using a NetAmps 300 system from Electrical Geodesics Inc. (EGI, Eugene, OR, USA) with the help of Netstation 4.5 software from the same company. The suppression of the alpha band during correct trials at site F3 was observed and it relates to learning. Additionally, children under seven had difficulties learning the concept of the reflection law.

7. The exploration of the effectiveness of an augmented reality (AR) application to assist mobile users in learning mobile photography concepts is presented in [17]. Emotional state indexes for engagement (ENG), relaxation (MED), interest (INT), and focus (FOC) are measured from the EEG of the users of this app. Twenty-eight participants (seven m. and twenty-one f.) of m. age = 20 were divided into two groups. An Emotiv EPOC+ is used for this study with 128 Hz sampling frequency and EEG processing was performed by the EmotivPro software which also extracts the emotional state indexes from the raw data. As it is mentioned in the Emotiv knowledge base [18], ENG is characterized by increased physiological arousal and beta waves along with attenuated alpha waves. INT is the degree of attraction or aversion to the current stimuli, MED is a measurement index of the ability to switch off and recover from intense concentration, and FOC is a measurement index of the fixed attention to one specific task. The effectiveness of the AR-based mobile photography application (ARMPA) compared to using a typical 2D mobile application is observed and verified by the results of the pre–post tests and also by measures of cognitive load [72] and the emotional state indexes mentioned above. According to these, students expend the least mental effort when using the ARMPA and they are more engaged to the learning task.

8. The potentialities and the issues concerning the use of portable EEG devices in educational contexts is presented in [73], where a case study is performed with a group of third-grade primary school students. The disruption of everyday life in the classroom, the collaboration between educators and researchers and the balance between budget, ease of equipment preparation, and usefulness of the collected data is discussed. Also providing an overview of recent studies using low-cost EEG devices, the paper describes a study conducted with a group of 17 third-grade primary school children aged between 8 and 9 years (ten girls and seven boys) in a regular school with which the researchers collaborated. Familiarity between researchers and educators and participants is very important in these contexts. Two baseline recordings of EEG activity were made: two minutes with eyes closed and two minutes with eyes open. The brain activity of the students listening to different explanations of mathematical applications and performing some arithmetic tasks was collected. Four types of portable devices were used: (i) Brainlink Pro, 512 Hz, two dry sensors, Bluetooth connection to Lucid Scribe software; (ii) Emotiv Epoc, 14 channel EEG semi-dry sensors, 128 Hz, data collected with Emotiv TestBench software; (iii) Epoc Flex, 32 channels, 128 Hz, passive Ag/AgCl gel sensors mounted on a neoprene cap, with EmotivPro software for data collection and analysis; and (iv) The Muse, four channels of dry contact electrodes from frontal and tempo-parietal lobes areas, 250 Hz, Mind Monitor application for data collection. EEGLab was mainly used for the EEG data analysis. As the main purpose of this study was to examine the issues and the methodology for EEG analysis in classroom settings, the sensations the students had with the devices were also collected with questionnaires.

9. The effects of context and gender in physics problems were investigated in [74]. The study focused on triggered situational interest. Being closely related to engagement, situational interest has behavioral, cognitive, and affective dimensions which were measured using psychophysiological tools including pupillometry, electrodermal activity (EDA), facial expression processing, and electroencephalography. The results were obtained by combining the information from each measurement, with pupillometry and EEG relating to cognitive engagement, EDA to arousal and emotional engagement, and facial expressions to emotional valence, respectively. Regarding EEG, the quotient of beta and the sum of alpha and theta power spectral density (PSD) in F3, F4, O1, and O2 scalp sites were employed for generating cognitive engagement indexes (CEI) every second, averaging over Hanning sliding windows from the last 20 sec. Sixty participants (32 f. and 28 m. of m. age = 23.7) were asked to read and solve 21 physics multiple choice questions of three different types depending on the kind of context they used: seven with a technical (T) context, seven with a humanistic (H) context, and seven with no context (N). A BrainVision BrainAmp with 32 channels with ActiCap active electrodes and the BrainVision Recorder software were used. Experiments were carried out in a lab environment and the surprising result (as the authors mentioned) that decontextualized problems (N) produced significantly higher performance than the other kinds (H and T), was found.

10. The levels of attention measured when a binaural wave stimulus was being heard by the students were examined in [75]. This study had two participants, science department students aged in the range from 20 to 28 and having a similar degree of knowledge. The Neurosky Mindwave portable EEG device was used, and data processing and analysis were performed by the accompanying Effective Learner application. It was observed that a frequency induction of a binaural wave at 10 Hz helped to increase the levels of attention during tasks such as viewing a video and reading a text. The duration of the EEG recordings varied from 2 min to 7 min.

11. The differences and similarities in the brain responses when performing readings and tasks in digital and paper textbooks were investigated in [76]. Apart from EEG they also collected eye tracking, galvanic skin response (GSR), and facial expression analysis (FEA) data. An emotive 14-channel portable device was used (probably EPOC-X, although it is not mentioned specifically) and the authors used the final metrics provided by the dedicated software of the devices without further processing. The sample consisted of three participants, two girls and a boy, in the sixth grade of primary education (around 12 years old) from different schools. Although the results obtained were mostly similar for both types of textbooks, a preference for the paper textbook was noticed in one participant. This was indicated by the frontal alpha asymmetry (FAA) metric, which was positive and related to the focus on, liking for, and attachment to the textbook.

12. The frontal ERP P300 asymmetry (fP3As) was investigated as a possible biomarker of cognitive control abilities related to judging performance during school days (Mondays and Wednesdays) and times (morning and afternoon) in [77]. This was to reveal important aspects of students learning such as school starting times, testing times, and sleep/nap and break duration guidelines to optimize student performance. The participants were 24 female high school students in southwestern Canada with m. age = 16.9 years and they performed a Stroop-like word–color judgement task randomly assigned to two groups (morning or afternoon test groups). The words were created with the Stim2 software and reaction times (RTs) and accuracy were recorded for each trial. The fP3A was determined via the subtraction of the P300 amplitude in electrode F3 from the one in F4. EEG data were recorded with Neurosoft, Inc. EEG with nine electrodes, and they were amplified and digitized with a SynAmps2 EEG system (Compumedics Neuroscan) at 1000 Hz using the SCAN 4.3 software. High-pass filtering at 0.15 Hz and 100 Hz low-pass filtering were applied. In summary, behavioral, and neural evidence was found to relate congruence judgement especially in the mornings, supporting a misalignment between adolescent circadian cycle and school start time.

13. Inhibitory control in negative priming (NP) is the subject of the research in [78]. The concept of NP is that if a stimulus has been previously ignored on purpose, subsequent reaction to the same stimulus would be impaired. This can cause a slower response time (RT) or a higher error rate. In [78], the process under investigation is the use of heuristics to solve fraction comparison tasks where one needs to think that a larger denominator leads to a smaller fraction and needs to inhibit the “larger natural number -larger fraction” strategy. Twenty-eight undergraduate students participated in the project with m. age = 20.8 years and none of them were math majors. The metrics used were the N1, N2, and P3 ERPs which are related to inhibition control. EEG data were recorded from 64 channels using the Brain Products (Germany) apparatus. Six electrode sites (Fz, F3, F4, Cz, C3, and C4) were used for N1 and N2 and nine sites (Fz, F3, F4, Cz, C3, C4, Pz, P3, and P4) for P3. The analysis of the data was performed with Brain Vision Analyzer 2.0. The study revealed that adults still needed to inhibit misleading strategies when performing fraction comparisons.

14. Again, the role of misconceptions, while studying biological phenomena this time, was examined in [79]. Inhibitory control plays an important part in this procedure where students must suppress tempting but inaccurate answers. The persistence of the “moving things are alive” heuristic into adulthood was the subject of the current research. Twenty-eight undergraduate students were recruited (13 f. and 15 m., m. age = 23.7 years). Images of intuitive (I) and contra intuitive (CI) drawings where the participants had to select the ones depicting living beings were displayed to the participants. EEG was recorded with Brain Vision Acticap, 64 ch. Stimuli were presented using the E-prime 2.0 software and data were analyzed using Brainvision analyzer v2.0. The N2 and LPP (Late Positive Potential) ERPs were used as metrics. The results indicated that identifying the living thing in the CI condition took significantly more time than in the I condition, and that the N2 and LPP components were greater in the CI conditions. This is an indication of the suppression of the heuristic that must be applied before the decision.

15. Intuitive thinking and analytical thinking were the subjects of the research in [80]. The former is mentioned as System 1 and the latter as System 2 in the bibliography. A simple task such as repeating four one-digit numbers, adding either zero or one to them, was employed for the EEG and pupillometry measurements. Thirty undergraduate students (m. age = 22.8, twenty-two f. and eight m.) were recruited for the research. The Psychophysics MATLAB toolbox was used for the experimental task and EEG data were recorded with an ActiCAP (Brain Products) 64 ch layout with ActiCHamp using Brain Vision software (v.1.10). EEGLAB and R language were also used for statistical analysis and graphical representations. The metrics used were the alpha and theta activity in parietal and frontal areas, respectively. It was demonstrated that cognitive control, working memory, attention, and long-term memory are involved in System 1 and 2, demonstrating an increased parietal alpha (at CPz) for the former and a decreased frontal theta (at Fz) for the latter. These results are in accordance with previous research findings.

16. An investigation related to the stress, attention, interest, and engagement levels in onsite and online higher education is presented in [81]. Eye tracking, galvanic skin response, and EEG were used in a sample of 20 postgraduate students (ages 22–25, 50% male and 50% female). The level of the brain waves was used for the metrics with the variation in the β wave correlated with attention while α is strongly correlated with meditation. Commitment, which is an indicator of motivation, was reflected in the β/(α + θ) index. The Emotiv EPOC+ was used for the EEG measurements with EmotivPRO v.2.0 software. Statistical analysis was performed with R language (v.3.6.3). The results of the research demonstrated that onsite learning is related to greater attention, interest, and emotional intensity levels, although stress levels are higher.

17. The differences in brain activity whilst performing various types of throwing games in children was explored in [82]. The power spectral densities of the brain waves were compared in three types of games: (i) throwing to a goal, (ii) throwing simultaneously with another opponent to the same goal, and (iii) just throwing. Eight children volunteers (four boys and four girls, m. age = 7.2) participated in the experiments. EEG recordings were made using a portable Emotiv EPOC-X headset with 14 recording channels and two reference electrodes. Artifact cancelation and a notch filter (at 50 Hz) were applied by the device. The Emotiv Brain Activity Map and Emotive TestBench applications were employed for the spectral analysis. Further pre-processing and ICA decomposition were performed by EEGLAB. The most significant differences in brain activity among the three types of games were observed in beta oscillations. The goal and simultaneous, (i) and (ii), types of games presented significantly higher values in beta band power spectral densities compared to just throwing. At the same time, the goal case exhibited significantly higher values than ‘simultaneous’ in the same band, indicating the association of beta waves with decision-making processes.

18. An effort to monitor the cortical activities of children and adults while completing complex cognitive tasks in the form of Schulte tables is presented in [83]. In their research, 12 children (three f. and nine m., aged 7–8) and 10 adults (three f. and seven m., aged 18–20) participated in the experiments. The tasks were considered difficult for the first group and easier for the second group due to the age and the relevant skills difference. Visual search, working memory and mental arithmetic were employed for the tasks. The ActiCHamp (Brain Products) EEG amplifier and ActiCap electrode sensors with 32 channels were used for EEG recording. The EEGLAB and Fieldtrip MATLAB toolboxes were used for preprocessing and wavelets analyses. Response times and brain bands (δ, θ, α, β, and γ) power spectral densities were used as metrics for the performances. The use of different problem-solving strategies (e.g., procedural vs. fact-retrieval) according to the age groups and experience gained was indicated by the results, which was trying to shed light on brain development and age-related changes in cognitive processes.

19–20. The role of the reward positivity ERP (RewP) and whether this component reflects an underlying learning process was the aim of the research presented in [84]. Thirty undergraduate students (twenty-three f. and eight m., mean age = 20) participated in the research where they had to learn to predict a disease type based only on health indexes information and positive or negative feedback upon correct or incorrect predictions, respectively. The experimental setup included the registration of responses to stimuli controlled by the Psychophysics toolbox. EEGs were recorded using a 64-channel ActiCAP and ActiCHamp amplifier (Brain Products) and analyzed with Brain Vision Analyzer software (version 7.6). According to the researchers, RewP reflects reward learning systems in the brain as its amplitude is diminished with learning. The same research question about the role of RewP is examined in a similar study by mainly the same team of researchers in [85]. In that research, a computational model and 30 human participants learn 60 words of a novel language.

21. Whether or not action is required to elicit a RewP and the impact of cue, choice, and action on its amplitude is investigated in [86]. Twenty-six students (13 m. and 13 f., m. age = 21, 54) participated in the experiments, where they completed four versions of a computer-based guessing game with different degrees of choice for the winning actions. EEG data were collected and amplified by ActiCHamp (Brain Products) and preprocessing was done in Brain Vision Analyzer (version 2.1.2). EEGLAB was also used for the analysis of the data. The results indicate that agency, which is defined as the sense of control over our actions and their outcomes, affects the generation of RewP, supporting the existence of a reinforcement learning (RL) system in the human brain.

22. The cognitive activities and performance on complex tasks were investigated in [87]. Cognitive load metrics were acquired from twenty-three university students (10 f. and 13 m. aged from 18 to 23, m. age = 19.25 and s.d. = 1.19) while performing listing and concept mapping tasks related to sustainability issues such as climate change, food systems, renewable energy, or water availability. Subjective behavioral metrics based on the NASA Task Load Index (NASA-TLX) and the Systems Thinking Scale Revisited (STSR) questionnaires were collected along with EEG measurements from the B-Alert X10 EEG system by Advanced Brain Monitoring (ABM). The cognitive load was estimated using the alpha/theta waves energy ratios on a second-to-second basis.

23. A dataset of parallel EEG recordings between subject pairs is presented in [88]. The public availability of synchronized EEG data is very important and can provide a platform for experimentation and benchmarking for hyperscanning and new processing algorithms exploring temporal, spatial, spectral, and functional synchronization aspects [89]. The dataset includes recordings of 16 subject pairs of one female and one male aged 18–24 and consists of four sections for each subject. Two sections of 1 min each correspond to the resting states of eyes open and eyes closed and two sections of 10 min each correspond to the parallel performance of a collaboration (solving a 100-piece puzzle) and a competition task (playing a domino game). Half of the recordings took place face to face and half took place online and they included four channels with signals from A1, A2, C3, and C4 electrode sites and they were obtained using a pair of Enophones (Eno, Montral, CA) wearable EEG devices and EEGLAB was used for the preprocessing.

**Table 1:** Neuroeducation projects details.

Id	Project	Subject	Sample	EEG Metrics	H/W Used	S/W Used	Wireless	Issues Addressed
1	Anderson et al. (2018) [62]	Efficacy of Just in Time teaching in anatomy education	24 students (11 m. + 23 f.), m., age = 20.74	N250 and RewP ERPs	BP * ActiCAP Xpress + 16 ch V-Amp	Psychophysics Matlab Toolbox, Brain Vision Recorder	No	Detailed examination of learning and retention with pre-class exercises. ERPs in accordance with expected behavior.
2	Anderson et al. (2023) [63]	Diagnostic accuracy factors examination	26 students (23 f. + 3 m.), m. age = 19.8, and 9 experts (6 f. + 3 m.), m. age = 44.1	RewP and N170 ERPs	BP 32 ch ActiCAP slim + LiveAmp	Psychophysics Matlab Toolbox, Brain Vision Recorder	Yes	Was diagnostic accuracy due to image recognition or acquisition of diagnostic expertise? ERPs in accordance with expected behavior.
3	Doukakis et al. (2019–2020) [64,65]	Students’ performance in programming tasks	8 first-year students (3 f. + 5 m.)	EEG negative means value and time	BIOPAC MP150, 2 ch used (C4-P4)	AckKnowledge 4.3	No	Differentiated teaching is required in programming. Limitation of small sample size.
4	Fard et al. (2020) [67]	Transfer learning during computer programming tasks	8 male students (18–21 y.o.)	EEG alpha and theta band power spectrums and models of neuronal connectivity	Emotiv EPOC-X, 14 ch	OpenSesame, EEGLAB, NeuCube with Spike Neural Networks (SNNs) models.	Yes	How to facilitate activation of prior knowledge. Cognitive load and prior knowledge. Use of neuronal activity patterns to interpret brain activity.
5	Poulsen et al. (2017) [68]	Brain synchronization and engagement while watching videos	42 f. (m. age = 22.4)	Intersubject correlation (ISC)	Emotiv EPOC+, 14 ch	Corrmap Matlab Toolbox	Yes	Measure inter-subject correlation with portable low-cost equipment.
6	Schroer et al. (2020) [71]	Spatial reasoning in children	21 children (11 m. + 10 f.), 6–12 y.o.	N2 and P3 ERPs, joint time frequencies of alpha band	NetAmps300 (EGI), 64 ch	Nestation 4.5 (EGI)	No	Can a simple educational video game be used to get children to learn the law of reflection? Suppressed alpha band after learning is confirmed. Limitation of exact localization of brain waves (inherent in EEGs)
7	Zhao et al. (2023) [17]	Effectiveness of AR app to assist learning	28 persons (7 m. + 21 f.) m. age = 20	Emotional state indexes from brain waves	Emotiv EPOC+, 14 ch	EmotivPro	Yes	Emotional state indexes for engagement, relaxation, interest, and focus are used for checking the effectiveness of using an AR app to learn photography. Limitations due to sample size and structure. Familiarization with experiments and tools is desired.
8	García-Monge et al. (2023) [73]	Exploration of potentialities and issues using portable EEG in classrooms	17 primary sch. children, (10 f. + 7 m.), 8–9 y.o.	2′ EEG recording with eyes closed and 2′ with eyes open. Frequency domain analyses.	Brainlink Pro (2 ch), Emotiv Epoc (14 ch), Epoc Flex (32 ch), Muse (4 ch)	EEGLAB, Lucid Scribe, Emotiv TestBench, EmotivPro, Mind monitor	Yes	Examine the methodological aspects for EEG analysis in the classroom.
9	Bouhdana et al. (2023) [74]	Effects of context and gender in physics problems	60 participants, (32 f. + 28 m.), m. age = 23.7	Cognitive engagement indexes β/(α + θ)	BP ActiCAP + 32 ch BrainAmp	BrainVision Analyzer (v2.0), Matlab	No	Multisensor data. EEG is employed for measuring cognitive engagement while solving problems in physics. Is context related to engagement?
10	Bos et al. (2020) [75]	Effects of low frequency binaural waves on attention levels	2 students with ages from 20 to 28 y.o.	Frequencies analysis performed by the proprietary software	Neurosky Mindwave, 2 ch	Effective Learner app. (Neurosky)	Yes	Attention levels are measured while subjecting the brain to low frequency (10 Hz) binaural waves. Limitation: very small sample size.
11	Onieva et al. (2021) [76]	Brain response differences and similarities while reading digital or paper textbooks	3 pupils (1 m. + 2 f.), 12 y.o.	Emotional state indexes from brain waves and FAA	Emotiv EPOC-X, 14 ch	Emotiv software	Yes	Multisensor data. EEG employed in commitment, interest, attention, stress, and relaxation estimation. Limitation: very small sample size.
12	Byczynski and Angiulli (2024) [77]	Congruence judgement in relation to school days and starting times	24 female students, m. age = 16.9	Frontal P300 ERP asymmetry	Neurosoft EEG quick caps (9 ch) and SynAmps2 (Compumedics Neuroscan) for amplification and digitization	SCAN 4.3 (Compumedics Neuroscan) and Stim2 for stimuli presentation	No	The frontal P300 ERP asymmetry from locations F3 and F4 is used as biomarker of cognitive control abilities. Variations in congruence judgement were observed, with lower levels in the mornings.
13	Fu et al. (2020) [78]	Inhibitory control performing fraction comparisons	28 students, m. age = 20.8	N1, N2, and P3 ERPs	BP ActiCAP, 64 ch	BrainVision Analyzer (v2.0)	No	Examines the negative priming effect (impaired reaction to stimulus that has been previously ignored). Adults still need to inhibit initial reactions in fraction comparisons.
14	Skelling-Desmeules et al. (2021) [79]	Persistence of misconceptions in biological studies	28 students, (13 f. + 15 m.), m. age= 23.7	N2 and LPP ERPs	BP ActiCAP, 64 ch	BrainVision Analyzer (v2.0), E-prime	No	Inhibitory control to initial decisions when seeing images of moving (or not) and alive (or not) things. Suppression of “moving thing is alive” heuristic. Suppression of counter intuitive (alive but not moving) information results in longer reaction times and differences in N2 and LPP ERPs.
15	Williams et al. (2019) [80]	Intuitive and analytical thinking	30 undergrad students (22 f. + 8 m.), (m. age = 22.8)	Alpha and theta activity in parietal and frontal areas	BP ActiCAP, 64 ch + ActiCHamp	BrainVision (v.1.10), EEGLAB and R language	No	Neural signatures for intuitive (System 1) and analytical (System 2) thinking are explored. System 1 is characterized by an increase in parietal alpha power. System 2 is characterized by an increase in frontal theta power.
16	Juarez-Varon et al. (2023) [81]	Analysis of stress, attention, interest, and engagement in onsite and online learning	20 pg students (22–25 y.o., 10 m. + 10 f.)	Alpha, beta, and theta activity	Emotiv EPOC+, 14 ch	EmotivPRO (v2.0) and R language	Yes	Brain wave power was used for estimating the levels of stress, attention, interest, and engagement. Commitment was also reflected in the variation in the brain waves’ power. Onsite learning related to higher levels of these indexes.
17	Garcia-Monge et al. (2020) [82]	Brain activity differences in various types of throwing games	8 children (m. age = 7.2)	Brain band (mostly beta) activity	Emotiv EPOC-X, 14 ch	Emotiv Brain Activity Map and Emotiv TestBench, EEGLAB	Yes	Higher β wave EEG power levels due to higher demand for motor control and competition situations in games using a goal. Three types of games, two with a goal and the third just throwing a ball are examined.
18	Khramova et al. (2021) [83]	Differences in cortical activities of adults and children	12 children (3 f. + 9 m.) 7–8 y.o. and 10 adults (3 f. + 7 m.) (18–20 y.o.)	Brain band (mostly alpha and beta) activity	BP ActiCAP, 32 ch + ActiCHamp	EEGLAB and FieldTrip	No	Use of different problem-solving strategies (e.g., procedural vs. fact-retrieval) was demonstrated among adults and children. Visual search, working memory, and mental arithmetic were employed for the tasks.
19	Williams et al. (2018) [84]	Reflection of reinforcement learning in RewP ERP	30 students (23 f.+ 7 m.), m. age = 20	RewP ERP	BP ActiCAP, 64 ch ActiCHamp	BrainVision Analyzer	No	Reward positivity (RewP) ERP studied with prediction of disease types. RewP amplitude diminishes with learning.
20	Williams et al. (2020) [85]	Reward prediction errors as indications of learning processes	30 students (19 f. + 11 m.), m. age = 20	RewP ERP	BP ActiCAP, 64 ch ActiCHamp	Brain Vision Analyzer and Psychophysics Toolbox for stimuli presentation	No	Reward positivity studied in learning words of a novel language. RewP amplitude diminishes with learning.
21	Hassal et al. (2019) [86]	The role of control over actions in reward *processing*	26 students (13 m. + 13 f.), m. age = 21.54)	RewP ERP	BP ActiCAP, 64 ch ActiCHamp	BrainVision (v.2.1.2) and EEGLAB, Psychophysics Toolbox Extension for stimuli presentation	No	Is action required to elicit reward positivity? Agency (sense of control) affects the generation of neural prediction error signals.
22	Barrella et al. (2019) [87]	Measurement of cognitive load in concept-handling tasks	23 students (13 m. + 10 f.), m. age = 19.25)	Alpha over theta waves ratio	B-Alert X10 EEG (ABM), 9 ch	ABM’s B-Alert Live software	Yes	More effort when creating concept maps than listing tasks. Self-reported cognitive load for concept mapping appears higher than the EEG estimated.
23	Hernandez-Mustieles et al. (2024) [88]	Public dataset of parallel EEG recordings during collaboration and competition tasks	16 subject pairs of 1 m. and 1 f. (aged 18–24)	n/a	Enophones, 4 ch	EEGLAB	Yes	Publicly available synchronized EEG dataset. Recordings of 2 × 1 min + 2 × 10 min involving face to face and online collaboration (puzzle solving) and competition (domino) tasks.
24	Romo-De Leon et al. (2024) [90]	Public dataset of EEG and other physiological signals recordings during two teaching scenarios for humanities studies	24 students (10 m. and 14 f., aged 18–25, mean = 21.33, s.d. = 1.4)	n/a	OpenBCI Ultracortex Mark IV, 8 ch	OpenBCI GUI and EEGLAB	Yes	Publicly available EEG dataset. Recordings of 2 × 1 min + 4 × 3 min involving traditional learning and partially immersive learning experiences.
25	Jamil et al. (2024) [91]	Brain to brain synchronization during remote learning	10 students aged 18–28 and one instructor	Time series and frequency domain correlations	Unicorn Hybrid systems (8 ch, 250 Hz)		Yes	Cross-correlation of 10 *student–instructor* pairs’ EEG signals to produce synchronization percentages. Detections of patterns of similarity. Limitations: vulnerability to artifacts.
26	Grubov et al. (2024) [92]	Cognitive abilities assessment and feedback in the form of recommendations	60 pupils in two age groups, 9–10 and 11–12 years old, 36 boys and 24 girls	Alpha band power variance	BP LiveAmp (64 ch, 500 Hz)	EEGLAB	Yes	Neuroadaptation in the educational process. Assessment of cognitive ability types such as visual search, working memory, mental arithmetic, and combinations of them.

* BP: Brain Products GmbH (also as Brain Vision LLC in the U.S).

24. Another dataset of EEG and other physiological signals is published in [90]. It consists of 24 recordings of students (10 m. and 14 f., aged between 18 and 25 years old, m. age = 21.33, and s.d. = 1.4) engaged in learning experiences in humanities under two scenarios. One is traditional learning and the other is with partially immersive learning experiences. Along with the physiological data, a set of psychometric data was collected from the participants through questionnaires. An OpenBCI Ultracortex Mark IV headset was used for the EEG recordings at 250 Hz using eight dry electrodes located in positions FP1, FP2, C3, C4, P7, P8, O1, and O2 based on the 10–20 system. The OpenBCI_GUI (v.5.2.2) software and EEGLAB were used for processing the recordings. Four sessions lasting three minutes each were recorded from each participant, preceded by two minutes of resting state (eyes closed and eyes open for one minute each).

25. Brain to brain synchronization, or hyperscanning, during remote learning sessions was the subject of investigation in [91]. The participants were 10 undergraduate information technology students from the United Arab Emirates University, aged from 18 to 28 years old and an instructor. EEG data were captured using a wireless Unicorn Hybrid system by g.Tec of eight channels at 250 Hz. The channels corresponded to positions Fz, C3, Cz, C4, Pz, PO7, Oz, and PO8, attaching the ground and reference electrodes to the participants’ mastoids. The cross-correlation of ten *student–instructor* pairs’ time series EEG signals were produced, and synchronization percentages were calculated. Correlation matrices in the frequency domain were also produced and patterns of similarity were detected using KNN classification.

26. An open-loop neuroadaptive system is proposed in [92]. The aim of this system is to assess the cognitive abilities (CAs) of the students and then suggest methods for their enhancement. This process is performed sequentially in an open-loop manner. The system operates in five stages: experimental session, data processing and formation, verification, and the implementation of recommendations for CA enhancement. The types of the assessed CAs include visual search, working memory, mental arithmetic and ability to combine the above. Experimental studies of the proposed system were conducted using the LiveAmp EEG device (Brain Products) to register the brain signals using 64 channels at a 500 Hz sampling rate and EEGLAB for their preprocessing. The sample consisted of 60 young students of two age groups, 9–10 years old and 11–12 years old and there were 36 boys and 24 girls. A total of 15 participants were excluded for various reasons. Behavioral (correctness and time needed) and physiological characteristics (normalized alpha band power variance) were used for attention and fatigue. Electrooculograms (EOGs) extracted from EEGs were used for the latter.

The above project descriptions are summarized in Table 1.

**Figure 4:**
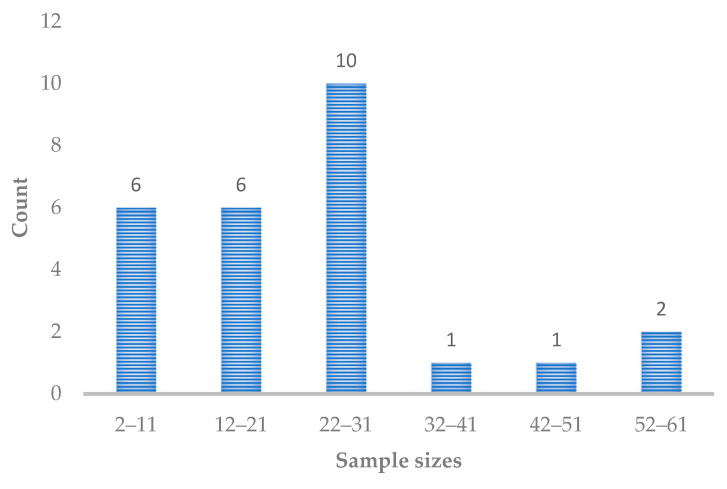
Counts of the sample sizes in groups of ten.

**Figure 5:**
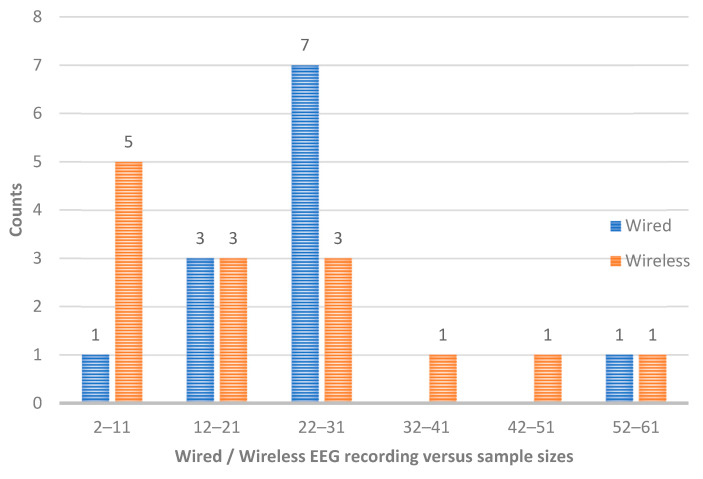
Counts of wired and wireless EEG recordings in each group of sample sizes.

**Figure 6:**
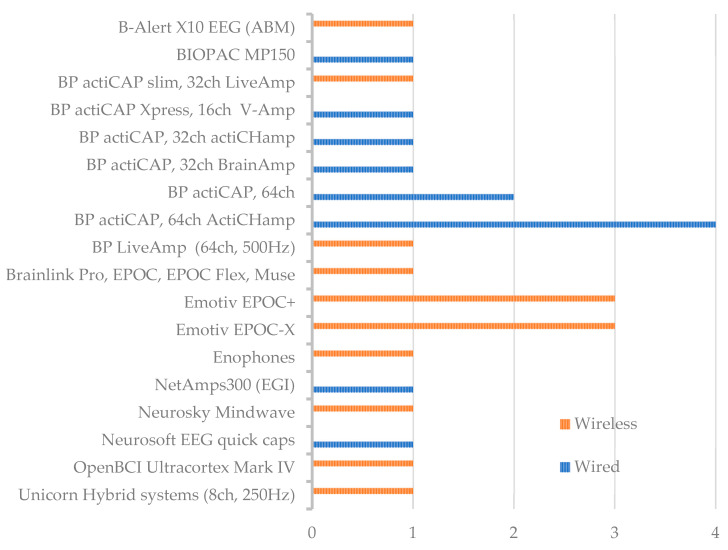
Counts of use of EEG devices and configurations (amplifier and caps).

In the following figures we can see the counts for each of the sample sizes in groups of ten (Figure 4), the count of times that wired and wireless EEG recordings were used in each group of sample sizes (Figure 5), and the count of times each EEG system was used (Figure 6).

**Figure 7:**
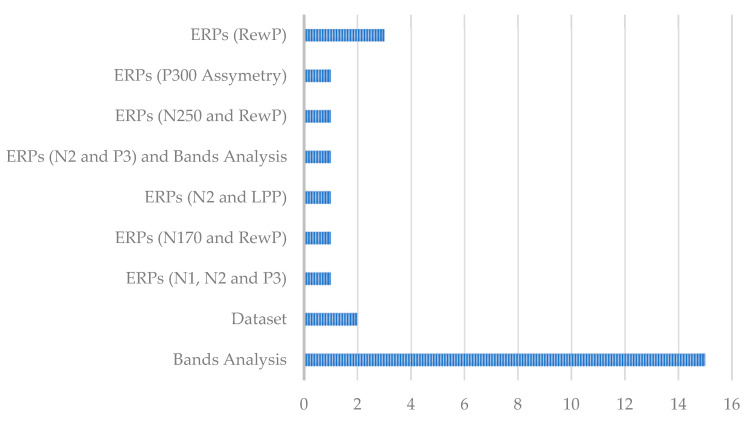
The way in which EEG signals were used in the presented projects.

The ways in which the EEG signals were used can be seen in Figure 7.

A discussion and further analysis of the neuroeducation projects that were presented above, organized according to the research questions set in the introduction, follows.

### 6.1. Q1: Methodological Aspects, Experimental Settings, Sample Sizes, Hardware, and Software Used

A challenge for neuroeducation, as presented in [2], is the lack of understanding between neuroscientists and educational researchers. Additionally, the highly controlled experimental settings used in neuroscience are not the typical natural environments where education is taking place. The availability of EEG apparatus, the comprehension of the relevant research methodologies, and the effectiveness of their applications in experiments are important factors in the bridging of these two worlds.

As mentioned in [73], some of the issues in neuroeducation research are the dominance of studies with university students while at the same time studies with primary school students are not conducted very often. This is mainly due to the organizational difficulties with studies involving pupils. However, learning at this period of life is much more vivid related to later stages of education [93] and it is worth arriving to a methodological framework allowing its study without disrupting everyday school life. This also brings up the issue of “naturalism” [94], regarding the research environment in controlled laboratory, partial naturalistic laboratory and naturalistic research. The latter is an ideal setting to register brain function while learning in natural conditions. However, it is the most difficult as well due to noise and interference problems, which are highly expected in these conditions. Low-cost devices can offer portability, wireless connectivity, and ease of use. These characteristics make them ideal for long-term studies in naturalistic environments, although the noise issues must be faced to a higher degree compared to more controlled environments. These issues exist in the projects that have been examined. The mean sample size was 23.32 ranging from two to sixty. The frequencies of the sample sizes in groups of ten are depicted in Figure 4, where it is shown that the most frequent sample size belongs to the zone 22–31 with 10 occurrences. At the same time, smaller sample sizes are more frequently met (twelve times) than higher sizes (four times). Regarding the age of the subjects, six papers describe projects with children. These are the projects with ids #6, #8, #11, #17, #18, and #26 according to the numbering in Table 1. Adult students are involved in the twenty other projects. That is, almost 75% of the projects involve students and adults in general.

Portable low-cost devices are used in 11 of the cases examined in Section 6 (Emotiv EPOC and Flex, OpenBCI, Neurosky, Enophones, Brainlink Pro, and Unicorn) while medium- to high-cost devices are used for the rest of the projects. Three projects with children use low-cost devices (#8, #11, and #17), so there are eight projects with adults that use low-cost devices as well. The EEG systems that are being used are depicted in Figure 6. In the same figure, it is shown whether the apparatus is wireless or not. Wireless equipment is being used in 14 cases and wired connections are used in 12 cases. From the six projects with children, wireless devices are used in four of them (Emotiv EPOC-X, Flex, Muse, Macrotellect Brainlink Pro, and BrainProducts LiveAmp). Half of the twenty projects with adults use wireless devices and the other half use wired ones. Wireless devices are used in all sample sizes but mostly in the group 2–11 as is shown in Figure 5. The selection of the EEG apparatus can depend on factors such as the budget of the project, the ease of use and comfortability (as mentioned specifically in the case of project #22), the target group (e.g., children or adults), the setting of the project, and the requirement for high signal quality. The final decision is often a compromise and a balance among these factors. It is very significant that a correct decision is made though because experiments should be made with as few measurements and repetitions as possible, especially when children are involved. The experience in the use of the apparatus is very important as well, as it is also mentioned in [73] describing project #8.

Together with the variety of low- to medium-cost EEG apparatuses to perform the experiments, there is also a variety of software performing the necessary signal preprocessing and analysis procedures. The availability of raw data can enhance these procedures with new processing methods and tools when open source and/or extensible software toolboxes and packages are used. It is therefore a very important characteristic when it is offered, as most of the low-cost devices prefer to pair with their proprietary software and provide this availability, of raw data, at an extra cost [48]. A combination of different software packages, depending on the level of analysis which is required in each case, was observed. Both open source and proprietary software is being used, the latter in combination with the corresponding EEG apparatus which is being employed in each case.

### 6.2. Q2: What Is Investigated, What Is the Subject of Research, and What Information Is Extracted from EEG Signals?

The subjects of investigation in the projects presented in this review belong to following categories:

*Efficacy of learning methods* (projects #1, #2, and #7) [17,62,63]. Establishing foundational knowledge with pre-class exercises is found to be effective in medical training as it is shown in project #1. The N250 and RewP ERPs were used as biomarkers in this project. Similar ERPs (RewP and N170) were used for project #2 as well, which was also about medical training. The source of diagnostic accuracy was investigated in this case and the question was whether it was due to image recognition capabilities or acquisition of diagnostic expertise. Active learning, where students learn by doing, provides a special experimental ground from which important conclusions can be derived regarding educational procedures. The use of virtual and augmented reality (VR and AR) tools in the learning process can also provide a very interesting experimental ground as it is shown in projects #7 and #24 [17,90].

*Students’ performance* (project #3) [64,65]. The need for differentiated education programs depending on learning skills and cognitive abilities was stated in this research which involved students of computer science in programming tasks both in visual and textual programming. The negative value of the means of EEG and the times needed to perform the tasks were used as metrics. The small sample size (eight students) is a limitation for this study, but it provides a solid ground for supporting this necessity.

*Brain synchronization* (projects #5 and #25) [68,91]. Attention and engagement as markers towards the “gestalt”, the feeling of a class as a distinct organization with its own “atmosphere” and mentality, can be derived using EEG technology [95]. Brain synchronization among the people involved in the teaching process (other students and instructor) is also very important. The “synergy zone” as is mentioned in [95], the ideal learning classroom environment, depends on factors such as engagement, attention, connection and enjoyment creating flow [96] and brain synchronization. The latter was investigated in projects #5 [68] and #25 [91], while a dataset of parallel EEG recordings is presented in project #23 [88] as is discussed below.

*The creation of datasets* (projects #23 and #24) [88,90]. Two projects created datasets for further processing. This is very important for the scientific community as it provides valuable data that can be used to develop, test, and benchmark new processing algorithms and techniques. As mentioned, the data were collected from parallel recordings in project #23 and regard collaboration and competition tasks between 16 subject pairs of one male and one female adult. The second dataset (project #24) was collected from two different teaching scenarios (traditional and partially immersive learning) in humanities studies.

*Examination of differences among different groups and factors* (projects #9, #10, #11, #12, #16, #17, and #18) [74,75,76,77,81,82,83]. The effects of context and gender in physics problems (project #9), the levels of attention when a binaural wave stimulus was being heard (project #10), differences and similarities when performing readings and tasks in digital and paper textbooks (project #11), judging performance during various school days and times (project #12), variability in stress, attention, interest, and engagement levels in onsite and online higher education (project #16), differences in brain activity while performing tasks towards a goal or in the presence of an opponent (project #17), and monitoring cortical activities of children and adults while completing complex cognitive tasks (project #18) are among the factors examined in these group of projects. Important conclusions and indications were found from the above projects as analyzed in their relevant descriptions in Section 6 and bands analysis provided the metrics in all of these, apart from project #12 which relied on frontal P300 asymmetry.

*Reinforcement learning and reward processing* (projects #19, #20, and #21) [84,85,86]. The reflection of underlying learning processes from the RewP ERP and its role in reinforcement learning was the subject of the research in project #19 where the field of medical training was used again, in project #20 where learning a new language was examined, and in project #21. In the latter case, the question was more complicated and regarded the necessity of action and the sense of control over actions to elicit a RewP.

*Cognitive load and abilities measurements* (projects #4, #22, and #26) [67,87,92]. Cognitive load and prior knowledge in the field of computer programming was investigated again in project #4. Methods to understand and interpret brain activity through neuronal connectivity and spike activity patterns were also demonstrated. Listing and concept mapping tasks related to sustainability issues provided the materials for the tasks in project #22. Cognitive load was estimated using the alpha/theta waves energy ratios. Neurofeedback, the self-regulation of brain activity, is a very promising approach, with important applications in addressing learning disorders [97]. An example of a system for the assessment of cognitive abilities and providing feedback in the form of suggestions for their enhancement was presented in project #26 [92] where an open-loop neuroadaptive system was proposed to assess and enhance cognitive abilities such as visual search, working memory, mental arithmetic, and their combinations.

*Forms of thinking and reasoning* (projects #6, #13, #14, and #15) [71,78,79,80]. Spatial reasoning related to the reflection law was investigated in project #6 where the use of a video game was employed for the learning task. Alpha band suppression during correct responses was observed while N2 and P3 ERPs were also used. Inhibitory control was the subject of the research in project #13 and the question was whether it is still used in adults to inhibit spontaneous answers to simple but tricky mathematical problems. The information used was obtained from EEG N1, N2, and P3 ERPs. Inhibitory control was again investigated in project #14 and the objective was to examine responses to intuitive and counter intuitive stimuli related to biological phenomena. Again, N2 ERPs were used together with the LPPs. Intuitive and analytical thinking, stated as System 1 and System 2, were explored in project #15. Activity in alpha and theta band waves in parietal and frontal areas was used for reaching conclusions.

*The examination of methodological issues for neuroeducation* (project #8) [73]. An investigation on the potentialities and the issues involved in neuroeducation projects in elementary schools was presented in project #8. The project described a case study with a group of primary school children and important highlights towards successful research strategies were discussed as described in more detail in Section 6.

Research in a multidisciplinary field such as the one of neuroeducation requires a team of specialists in the individual fields of education, neuroinformatics, psychology and cognitive science, and neuroscience. The EEG signal creates a large quantity of data which must be checked for validity and then undergoes a series of preprocessing and analysis steps before arriving in a condition from which interpretation and conclusions can be derived. The experimentation protocols must be carefully designed so that the data which will be collected will be the ones needed for the subject of investigation. To derive reliable conclusions from the EEG data, a proper combination of biomarkers should be used. ERPs are being used in nine of the projects presented in the previous section while 15 are based on EEG band processing to extract the features needed. Six of those projects involve children as subjects while ERPs are used additionally in only one of them (project #6). This might indicate a preference for bands analysis to provide biomarkers for projects involving children. Using band characteristics, ERPs, or connectivity features, machine learning can provide a set of tools to classify signals in proper states. However, behavioral information might also need to be combined with EEG data in neuroeducation. The ways in which EEG signals were used are presented in Figure 7.

As it can be pointed out, neuroeducation can offer the tools for better understanding the learning processes and the effects of using new educational methods, and offers the required expertise to optimize the learning conditions, as it happens with neurofeedback. New technological advances in the required hardware and software resources and the relevant methodological enhancements are opening a whole new horizon in this interdisciplinary field.

## 7. Conclusions

A scoping review on the use of EEGs in neuroeducation was presented in this paper. Some basic elements of what EEGs measure and what can be done with these signals were presented. Issues concerning the hardware which is required, focusing on portable, wireless, and low-cost solutions, were discussed. Some of the most common software solutions for the preprocessing and further analysis of the recorded signals were mentioned. A brief review of 26 recent neuroeducation projects, focusing on and discussing methodological aspects was provided as well. These projects were selected by applying the PRISMA statement to results returned by searching in PubMed, Scopus, and Google Scholar.

The analysis of the signals originating from the operation of the brain during educational tasks can offer invaluable insights and information regarding neurophysiological, cognitive, and educational aspects. The emergence of new technologies to facilitate signal collection through low-cost, wireless, and portable devices, and the variety of open software solutions to support the analysis of the results, can leverage research and new methodological approaches in neuroeducation and support the interdisciplinary cooperation of experts needed to accomplish research projects in this area.
